# A Case of Progressive Stroke on Posterior Circulation with Transient Bilateral Oculomotor Palsy

**DOI:** 10.1155/2018/1579426

**Published:** 2018-03-26

**Authors:** Chiaki Takahashi

**Affiliations:** Department of Neurosurgery, Takaoka City Hospital, 4-1, Takara-machi, Takaoka, Toyama, Japan

## Abstract

Infarction located in the midbrain and pons presents various ophthalmic symptoms, because of the damage of the nuclei that control the movement of internal and external ocular and palpebral muscles. We experienced a case which presented with rare ocular symptoms and course. A 61-year-old man presented with left hemiparesis and dysarthria, bilateral ptosis, and bilateral impaired eyeball movement: right eyeball movement was totally impaired and left could only perform slight adduction. MRI showed fresh stroke in the right thalamus, cerebral crus, and posterior lobe and cuneate lesion on bilateral paramedian portion of the midbrain. MRA showed occlusion in the P1 area of the posterior cerebral artery (PCA). Transesophageal echocardiography (TEE) showed findings of a patent foramen ovale (PFO). These findings suggested cardioembolic stroke as a cause of PCA occlusion and we prescribed rivaroxaban. The patient's eyeball and eyelid movement, only on the left side, was improved imperfectly 2 weeks later. We thought that neurological findings and course of this case may have arisen from dysfunction of the oculomotor nucleus and oculomotor fascicles, and MLF results from the presence of the lesion in paramedian midbrain and pons.

## 1. Introduction

We sometimes encounter patients with cerebral infarction in whom the main lesion is located in the posterior circulation. In such patients, especially in a case where the lesion is in the midbrain, the severity and symptoms show great variety depending on the location and range. In particular, the vascular supply in the midbrain is much more complex than in the pons and medulla. The vasculature is supplied by branches of three arteries: the posterior cerebral artery (PCA) and the basilar and the superior cerebellar artery (SCA), depending on the level [1]. The eponymic symptoms which represent oculomotor paresis with the paramedian midbrain lesion have been named Claude syndrome [2] and Benedict syndrome [3], and these definitions are also complex. In addition, Nothnagel reported symptoms of ipsilateral or bilateral oculomotor paresis and contralateral cerebellar ataxia due to a lesion in ventral side of the midbrain including the superior colliculus [4]. We think the present case is a rare case that presented transient bilateral oculomotor paresis with ptosis and contralateral ataxia and hemiparesis. The clinical symptoms suggested Nothnagel syndrome; however, we were not able to explain some points.

## 2. Case Presentation

**Figure 1:**
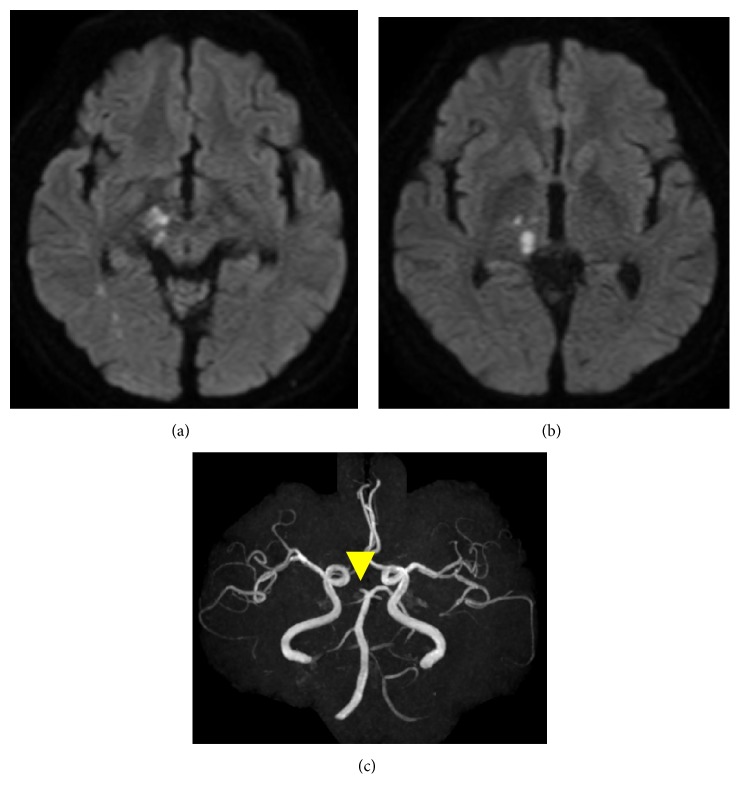
Head MRI performed about 24 hours after onset showed right occipital lobe, thalamus, cerebral crus, and midbrain fresh infarction (a, b). MRA showed right P2 (arrow head) and left SCA (superior cerebellar artery) occlusion (c).

A 61-year-old man with no remarkable history had never had a regular medical examination. He has been smoking about forty cigarettes per day for more than forty years. On the morning of the day before admission, he found that he was not able to read a book as his vision became suddenly distorted. He had additionally noticed weakness in his left leg during the daytime but went to bed without going to the hospital. The next morning, his weakness grew worse and he visited our hospital in a wheelchair due to gait disturbance. At this first admission, his NIH Stroke Scale (NIHSS) score was 4 points: hemiparesis 2, sensory disturbance 1, and dysarthria 1. He complained of diplopia when he looked to the right, but we could not observe his ocular deviation. His head MRI-DWI showed a scattered fresh infarction including the right occipital lobe, cerebral crus, and thalamus, and MRA showed occlusion of the P2 area of the right PCA and left SCA (Figure 1). Carotid ultrasound (CUS) showed findings of moderate arteriosclerosis, and plaque accumulation in the carotid bifurcation was not observed. He was admitted to our hospital. Electrocardiogram (ECG) and monitoring data showed no findings of arrhythmia. We speculated that the cause of this stroke might be atherosclerotic change at first. We decided to begin injection of argatroban hydrate and edaravone, and administered oral aspirin 100 mg.

**Figure 2:**
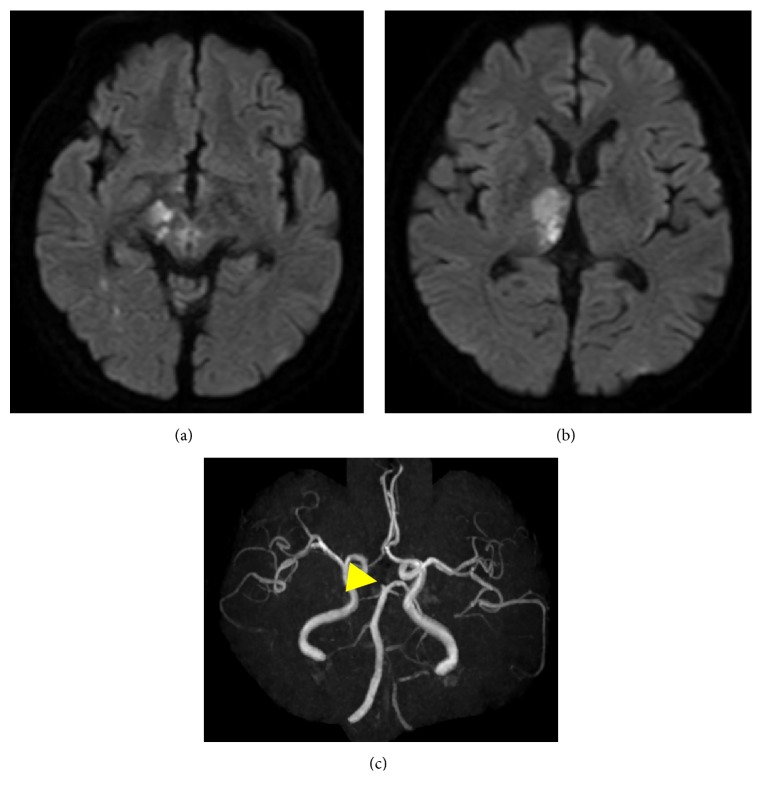
Head MRI performed after aggravation of neurological symptoms showed enlargement of infarction (a, b). MRA showed extension of occlusion (arrow head) to the P1 (c).

Early the next morning, a drop in consciousness level and aggravation of left hemiparesis presented. Emergency MRI was performed and showed enlargement of the lesion in the thalamus and additional cuneate fresh stroke on the right paramedian portion of the midbrain and small spotty fresh stroke on the left paramedian portion. MRA showed the extension of the occlusion of the PCA to the P1 portion (Figure 2). At this point, we thought perforators branching from proximal part of PCA were occluded due to the progression of thrombosis in the PCA. We added cilostazol to avoid extra thrombosis. After half a day, his consciousness level began to improve, and we performed a detailed neurological examination. At this time, we observed bilateral ptosis. He could not open his eyelids on his own and showed compensated contraction of the frontal muscle. His ocular position was median; right eyeball movement was totally impaired and left could only perform slight adduction with ocular nystagmus. The right and left pupil diameters were 6.0 mm and 5.0 mm. Light reflex was not observed on the right and was observed only slightly on the left.

During the course of treatment, transesophageal echocardiography (TEE) was performed to detect the cause of the stroke. It showed a patent foramen ovale (PFO) and noncompaction of the ventricular myocardium, which could have caused the stroke. According to the findings on TEE, we were convinced that the cause of the stroke was cardioembolic and changed his prescription from aspirin to rivaroxaban 20 mg.

**Figure 3:**
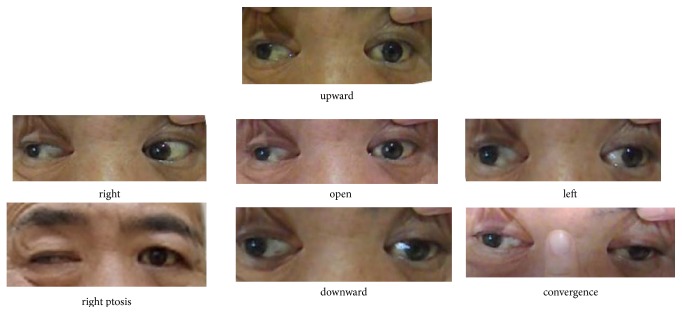
Findings of ptosis and oculomotor palsy one month after onset.

His consciousness level improved gradually, and oculomotor paresis including ptosis only on the left side was also improved imperfectly 2 weeks later. Finally, disturbance of upward and downward abduction remained on the left side and severe oculomotor palsy also remained in the right side (Figure 3). He could not achieve ocular convergence and Bell phenomenon in both eyes.

In addition, with regard to the motor paresis, we observed complete hemiplegia in the left upper extremity in the flexed position and mild hemiparesis in the left lower extremity. With the improvement of his consciousness level, he started standing and walking training with physical therapists. Although his muscle strength on the left lower extremity was sufficient, he had difficulty with those types of training due to ataxia of bilateral body trunk and lower extremities and attentional deficit as a higher brain dysfunction. Ultimately, he acquired walking ability with a cane, but he risked losing his balance and falling down when he had a lapse of concentration. With regard to higher brain dysfunction, mild cognitive dysfunction and memory deficit were present. At 12 weeks after initial presentation, he could leave our hospital and go home in relatively good condition.

## 3. Discussion

**Figure 4:**
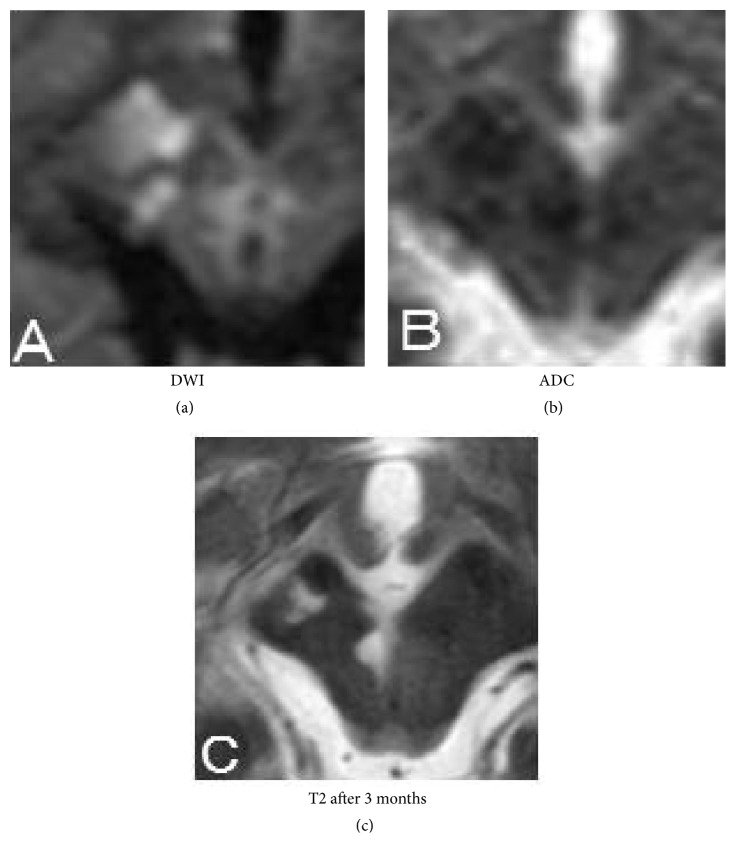
Head MRI on midbrain performed after aggravation of neurological symptoms. ADC map indicates the left paramedian lesion as stroke (a, b). Bilateral paramedian lesions three months after onset (c).

At first, neurological deficits in this case due to the occlusion of the P2 area of the PCA were not severe; they later became severe as a consequence of the enlargement of infarction due to the progressive thrombosis of the PCA and occlusion of the perforators branching from the P1 area. MRI showed a thalamic, cerebral crus and cuneate fresh stroke on the right paramedian portion of the midbrain and a very small spotty fresh stroke on the left paramedian portion. At first, we did not detect the left spotty infarction, but his clinical findings could not be explained; hence, we carefully reexamined his MRI referring to his apparent diffusion coefficient (ADC) map (Figure 4). The vasculature of the paramedian midbrain tegmentum was supplied by perforators branching like a fan from the P1 area up and down. These perforators are divided into 4 groups [5]. The first branch is called the thalamoperforating artery, and the second and third branches are the median mesencephalic arteries which supply the midbrain and the fourth branch is the superior pontine tegmental branch which supplies the pons [5]. Of these, the first and second branches bifurcate to the bilateral side with a probability of 10% [6]. In that case, the symptoms of the patient become potentially severe when the first or second branches are occluded by thrombus. It will bring the bilateral mesencephalic lesion. We detected the bilateral mesencephalic lesions in our case and considered that the second branch might be occluded.

When the patient's condition showed abrupt change at first, we observed these neurological symptoms: (1) bilateral total ophthalmoplegia with bilateral ptosis (the left eye could only perform slight adduction with ocular nystagmus), (2) contralateral hemiparesis and ataxia, and (3) consciousness disturbance. One month later, his neurological symptoms had improved: (1) right oculomotor palsy with ptosis, (2) contralateral abducens palsy with ocular nystagmus, (3) disturbance of bilateral suprainfraduction, (4) contralateral hemiparesis and ataxia, and (5) cognitive dysfunction and attentional deficit.

We considered the mechanism of the change of the symptoms and the affected region. At first, we could posit that the bilateral oculomotor palsy affected his symptoms because of the existence of the distinctive symptoms, bilateral ptosis, pupillary abnormality, and so on, and the lesion on paramedian midbrain lesion in MRI.

**Figure 5:**
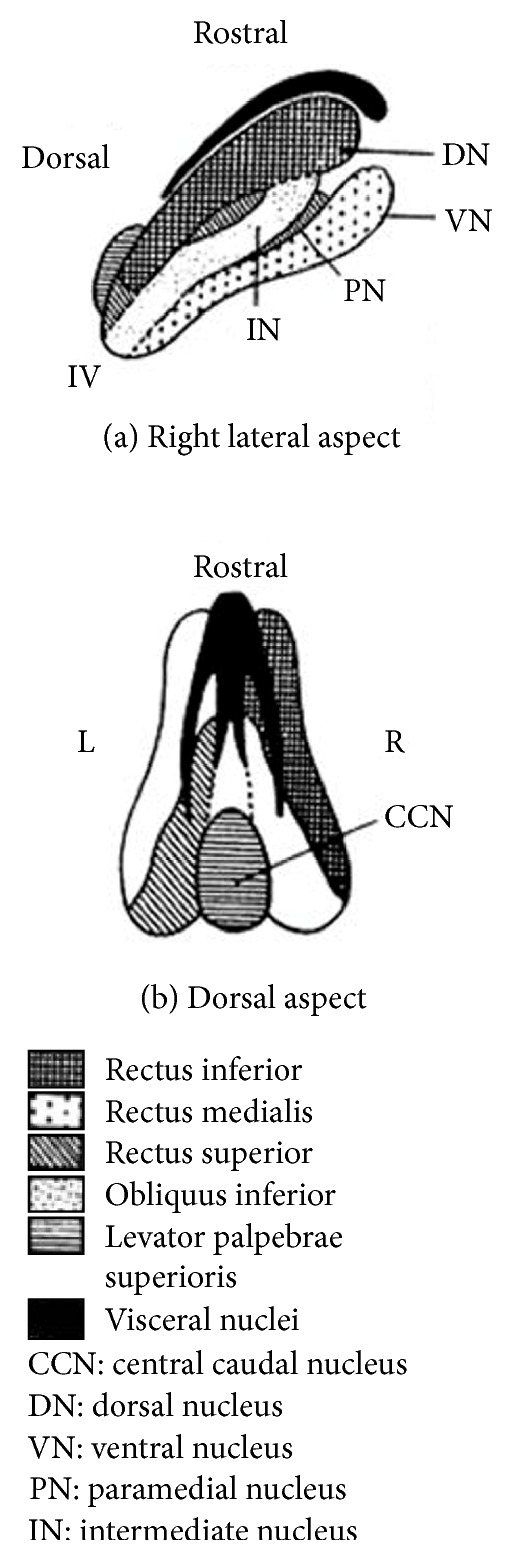
Structure of the oculomotor complex indicated by Warwick [7].

The nucleus of the oculomotor nerve is placed in the midline region of the midbrain. It exists in complex with multiple independent subnuclei, controlling the superior, inferior, and medial rectus muscle, inferior oblique muscle, levator palpebrae superioris muscle, and sphincter pupillae, respectively. Of all others, the caudal central subnucleus (CCN), which controls the levator palpebrae superioris muscle, exists in the center area of the midbrain and dominates the bilateral elevation of the eyelids [7, 8] (Figure 5). The nerve fibers which arise from the oculomotor subnucleus form the oculomotor fascicles near the red nucleus and make a transition to the extramedullary oculomotor nerve [9, 10].

At first, we inferred from his MRI and clinical symptoms that his partial neurological findings were due to nuclear damage of the whole oculomotor complex including the CCN. At that time, the severity of bilateral ptosis was almost the same between right and left eyelid. In addition, his pupil showed bilateral mydriasis. We thought that these findings are not likely with the damage of the oculomotor fascicles. However, left ptosis and oculomotor palsy were improved one month later. As a damaged CCN cannot later show unilateral improvement of ptosis [10], we determined that the CCN was not damaged and that part of the fascicles was damaged bilaterally and then showed mild or incomplete improvement on the left side. Indeed, a case of oculomotor palsy without ptosis was reported as a result of the sparing of the CCN from the damage of stroke [10]. Besides, it is said that the subnuclei of the superior rectus muscle control the contralateral side [11]. In our case strong disturbance of the supra and infraduction of the bilateral side persisted one month later. We speculated about the two possibilities to be able to evoke it. At first, according to the figure of the oculomotor complex by Warwick (Figure 5), we thought that the subnuclei of the supra- and infraduction were damaged bilaterally due to the involvement of the dorsal side of the upper to lower oculomotor complex in the infarction lesion. We thought that the bilateral nerve fibers of the levator palpebrae superioris muscle were damaged in the oculomotor fascicles and that of the other external and internal muscles controlled by the oculomotor nerve were damaged supranuclearly.

Secondly, ischemic lesion extended to the rostral interstitial nucleus of the medial longitudinal fascicles (riMLF) or interstitial nucleus of Cajal placed in rostral-medial side of the red nucleus might bring Parinaud's syndrome [12].

Then, we considered the behaviors which are impossible to explain only with the dysfunction around the level of the oculomotor nucleus.

The positions of the bilateral eyes were almost neutral at first and he could abduct only his left eyeball slightly with ocular nystagmus. One month later, his right eyeball was fixed completely to the abducted position and he could abduct his left eyeball incompletely with ocular nystagmus.

Granted that the stroke lesion in the upper pons which was impossible to detect by MRI existed, we considered several possibilities. One-and-a-half syndrome and medial longitudinal fascicles (MLF) syndrome are caused by the damage of paramedian area of the pons. The former does not fit into the behaviors of his eye movement, because he could adduct his left eye. Besides, we also considered WEBINO syndrome which is caused by the bilateral MLF syndrome. In such a case, we could observe the alternative exotropia when the patient holds fixation with a single eye. However his right eye always fixed into the abducted position regardless of the behavior of right eye; it also does not fit. Besides, it is reported that WEBINO syndrome due to pontine lesion often shows convergence impairment [13].

So, we speculated about it as follows.

At first, both bilateral oculomotor palsy which was stronger in the right eye and the right MLF syndrome existed, and then the left oculomotor palsy improved, and the right strong oculomotor palsy and the right MLF syndrome might remain finally. The left abducens palsy might be a movement of the intact side with nystagmus. Besides, his convergence palsy is explainable by coexisting dysfunction of the oculomotor nucleus.

**Figure 6:**
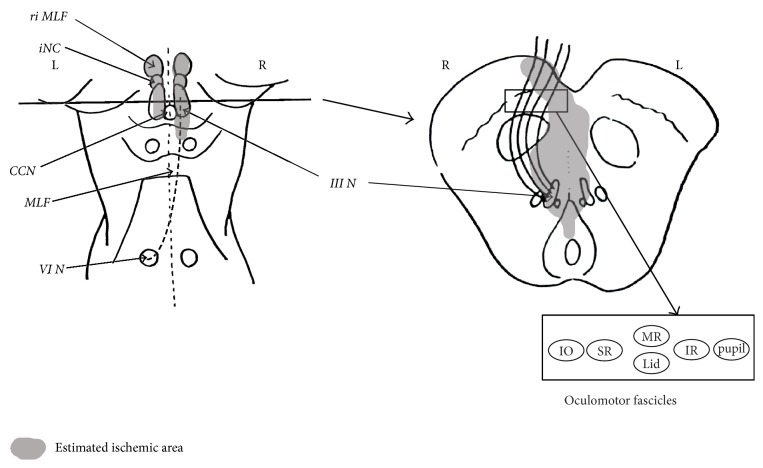
Picture in the left shows dorsal side of the midbrain and pons and in the right shows cross-sectional view of the midbrain along the line. Gray areas mark the ischemic area. riMLF: rostral interstitial nucleus of medial longitudinal fasciculus, iNC: interstitial nucleus of Cajal, CCN: caudal central subnucleus, MLF: medial longitudinal fasciculus, III N: oculomotor nucleus, VI N: abducens nucleus, IO: intraocular muscles, SR: superior rectus muscle, MR: medial rectus muscle, Lid: Levator palpebrae superioris muscle, IR: inferior rectus muscle, and pupil: sphincter pupillae muscles.

We described the final estimated ischemic area in his brain stem (Figure 6). The figure in the left side shows the dorsal side of the brainstem and it describes ischemic area including the bilateral oculomotor nucleus without CCN and right MLF. The figure in the right side shows cross-sectional view of the midbrain along the line. It describes the ischemic part of the oculomotor fascicles pointed by the MRI image. Castro et al. reported these fascicles lines like the schema in the lower right side [14].

In addition, cognitive dysfunction and attentional deficit were presented and may have arisen from the inclusion of the thalamus [15]. Contralateral hemiparesis may have arisen from the stroke of the cerebral crus, and ataxia may have arisen from the thalamic stroke.

We obtained informed consent from the patient to use personal information and data including photographs of eyes, images of MRI, and health records.
